# Effect of Liothyronine Treatment on Quality of Life in Female Hypothyroid Patients With Residual Symptoms on Levothyroxine Therapy: A Randomized Crossover Study

**DOI:** 10.3389/fendo.2022.816566

**Published:** 2022-02-22

**Authors:** Betty Ann Bjerkreim, Sara Salehi Hammerstad, Hanne Løvdal Gulseth, Tore Julsrud Berg, Lars Johan Omdal, Sindre Lee-Ødegård, Erik Fink Eriksen

**Affiliations:** ^1^Department of Endocrinology, Morbid Obesity and Preventive Medicine, Oslo University Hospital, Oslo, Norway; ^2^Institute of Clinical Medicine, Faculty of Medicine, University of Oslo, Oslo, Norway; ^3^Department of Endocrinology, Pilestredet Park Specialist Center, Oslo, Norway; ^4^Department of Pediatrics, Oslo University Hospital, Oslo, Norway; ^5^Department of Chronic Diseases and Ageing, Norwegian Institute of Public Health, Oslo, Norway; ^6^Department of Endocrinology, Balderklinikken, Oslo, Norway; ^7^Department of Transplantation, Oslo University Hospital, Oslo, Norway; ^8^The Faculty of Dentistry, University of Oslo, Oslo, Norway

**Keywords:** hypothyroidism, quality of life, liothyronine, levothyroxine, thyroid, deiodinase 2

## Abstract

**Objective:**

The effects of levothyroxine (LT4)/liothyronine (LT3) combination therapy on quality of life (QoL) in hypothyroid patients former on LT4 monotherapy have been disappointing. We therefore wanted to test the effects of LT3 monotherapy on QoL in hypothyroid patients with residual symptoms despite thyroid stimulating hormone (TSH) values within the reference range.

**Design:**

Female hypothyroid patients with residual symptoms on LT4 monotherapy or combination LT4/LT3 therapy received LT3 and LT4 monotherapy, respectively for 12 weeks in a non-blinded randomized crossover study.

**Methods:**

Fifty-nine patients aged 18-65 years were included. QoL was assessed using one disease-specific questionnaire (ThyPRO) and two generic questionnaires (Fatigue Questionnaire and SF-36) at baseline and at the end of the two treatment periods. Clinical indices of cardiovascular health (resting heart rate and blood pressure), as well as thyroid tests, were assessed at baseline and at the end of the two treatment periods.

**Results:**

After 12 weeks of LT3 treatment, 12 of the 13 domains of the ThyPRO questionnaire (physical, mental and social domains) showed significant improvements. The most pronounced improvements were less tiredness (mean -21 ± 26; *P*<0.0001) and cognitive complaints (mean -20 ± 20; *P*<0.0001). LT4 monotherapy exerted minor effects on two domains only (cognitive complaints and impaired daily life). All three dimensions’ scores in the Fatigue Questionnaire (physical, mental and total fatigue) improved after LT3 treatment compared to baseline (*P*<0.001), and in the SF-36 questionnaire 7 of 8 scales showed significantly better scores after LT3 treatment compared to baseline. There were no differences in blood pressure or resting heart rate between the two treatment groups. TSH in patients on LT3 was slightly higher (median 1.33 mU/L (interquartile range (IQR) 0.47-2.26)) than in patients on LT4 (median 0.61 mU/L (IQR 0.25-1.20; *P*<0.018). Five patients on LT3 dropped out of the study due to subjectively reported side effects, compared to only one on LT4.

**Conclusions:**

LT3 treatment improved QoL in women with residual hypothyroid symptoms on LT4 monotherapy or LT4/LT3 combination therapy. Short-term LT3 treatment did not induce biochemical or clinical hyperthyroidism, and no cardiovascular adverse effects were recorded. Further studies are needed to assess the long-term safety and efficacy of LT3 monotherapy.

**Clinical Trial Registration:**

ClinicalTrials.gov, identifier NCT03627611.

## Introduction

Hypothyroidism remains a significant clinical conundrum for clinicians. While the majority of patients on levothyroxine (LT4) therapy are asymptomatic, 5-10% of patients present residual symptoms despite normal thyroid tests (1). The main residual symptoms are pronounced cold-intolerance, fatigue, weight gain, cognitive impairment and mood disturbances (1). According to the available literature (2, 3), quality of life (QoL) is indeed reduced in patients with thyroid disorders. These symptoms persist despite adequate LT4 substitution judged by free T4 (FT4), free T3 (FT3) and thyroid stimulating hormone (TSH) levels. This led to the introduction of combination therapy with LT4 and liothyronine (LT3). This approach is based on the concept that hypothyroid patients with polymorphisms in deiodinase 2 (D2-Thr92Ala) (4) may exhibit reduced conversion of T4 to T3 resulting in reduced intracellular levels of T3 resulting in reduced activation of intracellular T3 receptors. Clinical studies assessing the effects of LT4/LT3 combination therapy have, however, been disappointing, with the majority of studies showing no significant differences in psychological or quality of life (QoL) scores compared to LT4 monotherapy in several meta-analyses (5–7). Only two research groups have reported superior results for combination therapy with LT4/LT3 with significant improvements in mood and psychometric tests (8–11). Several reasons for the lack of effect of combination therapy have been invoked; inclusion criteria for the studies, small sample sizes, short half-life of LT3 resulting in fluctuating hormone levels, and the fact that most patients were over-substituted with suppressed TSH (5, 12, 13). Furthermore, it has been stated that future clinical trials should include a more homogenous group of hypothyroid patients (14), and preferably patients with residual symptoms (12), in order to evaluate possible differential effects of treatment with LT4 and LT3.

Earlier studies have shown that treatment with LT4 monotherapy reduces FT3 concentrations in serum creating a high LT4/LT3 ratio (15–17). Lower serum levels of FT3 may imply a reduced availability of intracellular T3 in the brain and other tissues, despite optimal TSH levels in serum. We therefore hypothesized that LT3 monotherapy might achieve higher intracellular T3 levels, thereby causing possible improvements in QoL.

Skepticism towards treatment with LT3 in patients with hypothyroidism, and especially LT3 monotherapy, is based on the concern for increased risk of developing potential adverse effects on heart (18) and skeleton due to increased bone turnover (19), and the potential of wide fluctuations in serum hormone level as the LT3 half-life is less than 24 hours (20–22). However, Celi et al. examined the pharmacodynamic effects of monotherapy with LT3 in hypothyroid patients using a three times-daily regimen and achieved a steady-state (23). In addition, no significant differences were observed in cardiovascular parameters (24).

We have previously described the primary objective of a randomized crossover study evaluating female hypothyroid patients with residual hypothyroid symptoms using LT4 and LT3 monotherapy for 12 weeks each and reported the effect of LT3 monotherapy on skin temperature and activation of brown adipose tissue as the main outcome (25). As far as we know, there are no available data on the effect of LT3 on QoL in hypothyroid patients with residual symptoms and with TSH level within the reference ranges. The secondary objectives of this randomized crossover study were therefore to; 1) Compare disease-specific and generic; 2) Assess the association of D2-Thr92Ala deiodinase 2 polymorphisms and QoL perceived by patients; and 3) Monitor possible cardiovascular adverse effects.

## Materials and Methods

### Ethics

The study was approved by the Regional committee for ethics in medical research (ref. no. 2017/1883) and by the Norwegian Medicines Agency (ref. no. 18/02175). Written informed consent was obtained from all patients. The study was registered at ClinicalTrials.gov (NCT03627611) and performed in accordance with the declaration of Helsinki. The study was carried out between June 2018 and June 2020 at the Department of Endocrinology, Morbid Obesity and Preventive Medicine at Oslo University Hospital.

### Study Design and Patients

**Figure 1:**
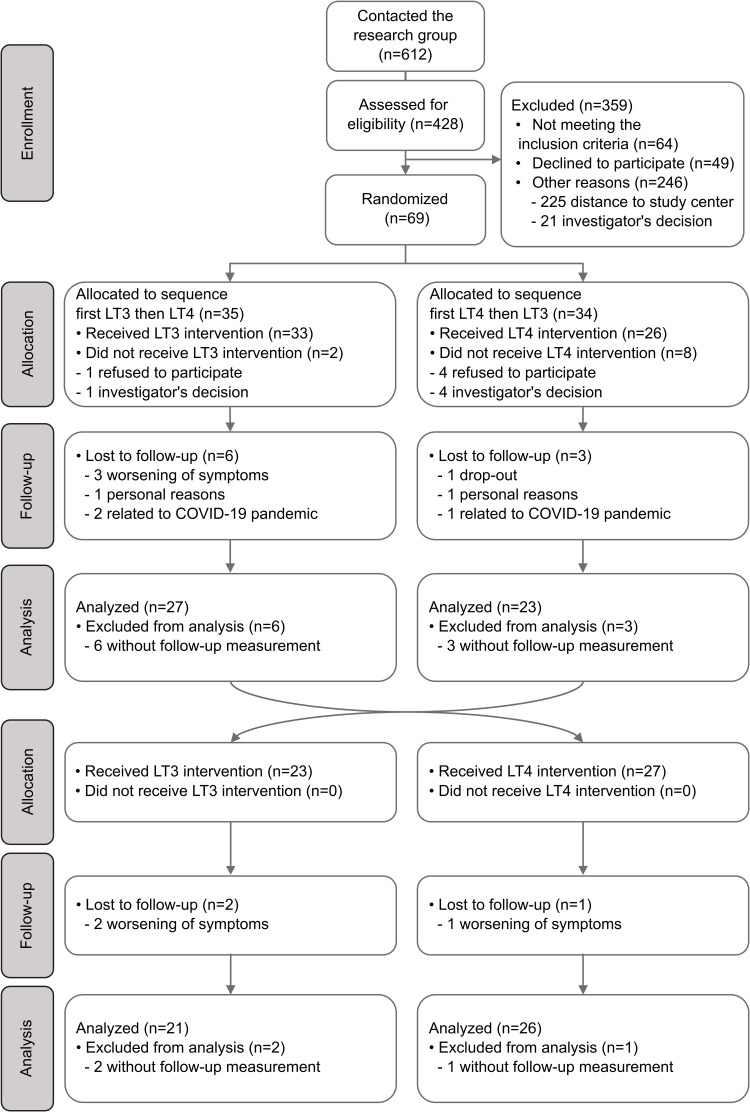
Flow chart describing recruitment, randomization and analysis of patients in the study.

Study design including recruitment process have previously been reported (25). In brief, sixty-nine female hypothyroid patients aged 18-65 years with residual hypothyroid symptoms despite optimal LT4 treatment were included and randomized in a non-blinded crossover design with LT4 and LT3 monotherapy for 12 weeks, followed by a crossover for another period of 12 weeks. However, **Figure 1**, which has been presented earlier (25), shows that ten patients dropped out before treatment initiation. Patients were recruited from the endocrine outpatient clinic at Oslo University Hospital and endocrinologists in private practice in Oslo. Computer-generated block randomization with randomly selected block sizes of 4, 6 and 8 was performed to assign patients to receive either LT4 or LT3 treatment first, before changing to the second treatment. The random allocation sequence was prepared in a set of sealed envelopes, before the specific treatment sequence for each individual participant was concealed at the start of the first treatment period.

Patients with diabetes or other endocrinological diseases, treatment with glucocorticoids or other hormones, pregnancy or breastfeeding, psychiatric diseases, or any other significant chronic or acute disease such as heart or kidney failure or liver cirrhosis were excluded. As earlier described (25), presence of residual symptoms was assessed by a ten-question form (**Supplementary 1**). If patients answered “yes” to at least three of the ten stated symptoms, they were reported to meet that inclusion criteria.

If a patient was already on LT3/LT4 combination therapy, LT3 monotherapy or desiccated thyroid extract before study start, they had to complete a 4-week run-in period on LT4 monotherapy. We estimated the LT4 dose by calculating 15 μg of LT3 as equal to 50 μg of LT4, based on a pharmacoequivalence study which showed that the LT4/LT3 equivalence ratio is approximately 3:1 (23). LT3 treatment was started at a dose of one third of the patient’s previous LT4 dose. LT4 treatment was maintained at the patient’s usual dose. A wash-out period was not included between the two treatment periods due to the shorter half-life of LT3 compared to LT4 and a relatively long treatment period of 12 weeks. After attending the initial study visit, patients were advised to take LT4 treatment once daily, half an hour before breakfast, and LT3 treatment thrice daily, half an hour before, or two hours after, a meal.

Patients were supplied with study drugs in boxes containing 100 tablets in total. They were asked to keep a diary for the administration of the study drugs and also bring all the boxes at the end of the current treatment period for evaluation and counting of tablets to ensure compliance. Adverse events were evaluated by standard medical history and physical examination including auscultation of heart and lungs, palpation of neck, abdomen and distal pulses, at baseline and after both treatment periods together with resting heart rate and systolic and diastolic blood pressure.

Thyroid function tests were performed every four weeks during the study period and treatment doses were adjusted, if necessary, aiming to achieve TSH levels 0.1-1.5 mU/L.

At the end of the study, all patients were asked about treatment preference.

A medical doctor (BAB or SLØ) performed the QoL assessment, and one study nurse measured blood pressure, resting heart rate, and performed the blood sampling.

### Laboratory Assessment

All blood samples were collected in a fasting state in the morning before medication was ingested, approximately 24 hours after the last LT4 dose and 14 hours after the last LT3 dose. All analyses were performed at the Hormone Laboratory at Oslo University Hospital.

TSH (reference range: 0.5-3.6 mU/L) was measured with non-competitive immunofluorometric analysis by Autodelfia (Wallac Oy, Turku, Finland). FT4 (8.0-21.0 pmol/L) was measured with solid-phase time-delayed fluoro-immunoassay with back-titration by Autodelfia (Wallac Oy, Turku, Finland). FT3 (2.8-7.0 pmol/L) was measured with competitive electrochemiluminescence immunoassay by Cobas e601 (Roche Diagnostics, Indianapolis, IN, USA).

### Analyzation of D2-Thr92Ala Polymorphism

A blood sample from all patients was collected in EDTA tubes at the baseline visit. DNA was extracted using the salting out method in combination with proteinase K. DNA concentration was assessed with a NanoDrop ND-1000 spectrophometer, (Promega, Madison, WI, USA). The TaqMan^®^ assay C:15819951 (Applied Biosystems, Foster City, CA, USA) were used to investigate the D2-Thr92Ala polymorphism.

### Quality of Life Assessment

Patients reported QoL by answering three different questionnaires (ThyPRO, SF-36 and Fatigue Questionnaire) at a baseline visit and at the end of each 12-week treatment period.

ThyPRO is a validated questionnaire and consists of 85 items grouped into 13 multi-item scales measuring physical, mental and social domains of functioning and well-being in hypothyroidism, hyperthyroidism, non-toxic goiter and Grave´s orbitopathy, and one single-item scale measuring overall impact of thyroid disease on QoL. Each of the 13 ThyPRO scales have a possible score between 0-100, with higher scores indicating poorer health status (26–29). Twenty-six of 59 patients answered an abbreviated ThyPRO questionnaire at the baseline visit, consisting of 39 questions, where all scale scores were transformed linearly to the corresponding 0-100 score in ThyPRO 85 according to a table.

The 36 items in SF-36 measuring physical and mental domains, are grouped into 8 multi-item scales including physical and social functioning, role emotional and physical, bodily pain, mental health, vitality and general health. Each scale is scored between 0-100, with higher scores indicating better health status (30).

Fatigue Questionnaire consists of 11 items divided into two main dimensions; physical fatigue (7 items) and mental fatigue (4 items). Combining the scores of physical fatigue and mental fatigue produce total fatigue as a third dimension, with a maximum score of 33. Higher scores imply higher levels of fatigue (31).

### Sample Size and Power Calculations

As previously described (25), a study population of 60 patients was set as the recruitment target in our study. This calculation was based on temperature data since the main outcome in our crossover study was dermal skin temperature and brown adipose tissue activation assessed by thermal imaging. However, based on evaluation of the hypothyroidism physical symptom scale in ThyPRO (26), with a minimal clinical difference of 10 and standard deviation of 16, we calculated that a sample size of 43 would be sufficient to detect a significant difference with 80% power and α = 0.05 between the two study treatments.

### Statistical Analyses

Data were analyzed by one-way ANOVA and paired *t* tests for variables with normal distribution, Wilcoxon signed rank test and Mann-Whitney U test for variables without normal distribution, and Chi-square test for categorical variables. All variables were tested for normality by quantile-quantile (QQ)-plots and histogram. Statistical significance was defined as two-tailed *P* < 0.05. All statistical analyses were performed using IBM SPSS Statistics for Windows, version 26 (IBM Corp., Armonk, N.Y., USA).

## Results

Fifty-nine patients were randomized and received intervention. Forty-seven (79.7%) completed both treatment periods. **Figure 1** has been presented earlier (25) and describes the flow of patients in the study. Six patients withdrew because of side effects and worsening of symptoms after receiving intervention, one on LT4 treatment and five on LT3 treatment. Side effects reported were (fatigue, dizziness, muscle aches, anxiety, irritability), without differences between LT3- and LT4 groups. One patient was lost-to follow up for unknown reason. Two patients withdrew due to personal reasons (one on LT4 and on one LT3) and three patients were unable to continue participation due to the COVID-19 pandemic (two on LT3 treatment and one on LT4 treatment).

The mean age of the patients was 42.9 years ± 9.7 and the mean duration of previous treatment with LT4 was 10.6 years (range: 1-28). Fifty-seven patients (96.6%) reported fatigue as a persistent symptom, despite TSH values within the reference range. Patient characteristics at baseline are presented in **Table 1**, and have been reported previously (25).

**Table 1:** Baseline characteristics.

	Prior to randomization (n = 59)	Allocated to first LT4 then LT3 (n = 27)	Allocated to first LT3 then LT4 (n = 32)	*P* value
Age at inclusion (years)	42.9 ± 9.7	42.8 ± 8.6	42.9 ± 10.7	0.991
Age at hypothyroidism diagnosis (years)	30.6 ± 10.2	29.8 ± 8.6	31.2 ± 11.4	0.590
Duration of substitution monotherapy LT4 (years)	10.6 ± 7.0	10.9 ± 7.3	10.3 ± 6.8	0.778
Type of therapy at inclusion				
LT4 monotherapy	46 (78.0)	20 (74.1)	26 (81.3)	0.513
LT4/LT3 combination therapy	12 (20.3)	7 (25.9)	5 (15.5)	0.728
Thyroid extract	1 (1.7)	0 (0)	1 (3.1)	1.000
Etiology of hypothyroidism				
Autoimmune/idiopathic	56 (94.9)	27 (100%)	29 (90.6)	0.299
Post-surgical	2 (3.4)	0 (0)	2 (6.3)	0.549
Radioiodine	1 (1.7)	0 (0)	1 (3.1)	1.000
Body mass index (kg/m^2^)	28.1 ± 5.6	28.5 ± 5.9	27.8 ± 5.5	0.624
Resting heart rate (beats/minute)	64.7 ± 11.2	67.4 ± 12.1	62.4 ± 9.9	0.084
Systolic blood pressure (mmHg)	118.0 ± 13.7	115.5 ± 13.2	120.2 ± 13.9	0.191
Diastolic blood pressure (mmHg)	78.3 ± 7.9	77.2 ± 8.7	79.4 ± 7.0	0.282
TSH (mU/L)	0.64 (0.26-1.60)	0.82 (0.28-1.50)	0.63 (0.25-1.9)	0.767
FT4 (pmol/L)	16.8 (14.7-19.0)	17.0 (14.2-20.0)	16.4 (14.8-18.0)	0.970
FT3 (pmol/L)	4.4 (3.8-4.9)	4.4 (3.8-4.8)	4.4 (3.8-5.0)	0.664
Positive TPO-ab^1^	31 (52.5)	17 (63.0)	14 (43.8)	0.226
Residual hypothyroid symptoms				
Fatigue	57 (96.6)	26 (96.3)	31 (96.6)	1.000
Cold-intolerance	54 (91,5)	26 (96.3)	28 (87.5)	0.460
Cognitive disturbances	48 (81.4)	24 (88.9)	24 (75.0)	0.303
Emotional disturbances	38 (64.4)	16 (59.3)	22 (68.8)	0.627

Data are presented as mean ± SD or number (%) or median (interquartile range: 25-75%) as appropriate.

TSH, thyroid stimulating hormone; FT4, free thyroxine; FT3, free triiodothyronine; TPO-ab, thyroid peroxidase antibodies. ^1^Cut-off value for positive TPO-ab was 35 kIU/l.

Median weekly (IQR 25-75%) doses of study drug administered were 775 µg (658–950) for LT4 and 245 µg (210–280) for LT3 (data not shown). On average, both the LT4 and LT3 dose had to be adjusted 0.7 times during the 12-week treatment period.

### Thyroid Function Parameters

TSH levels were kept between 0.1-3.6 mU/L in both treatment groups, except in five patients on LT4 treatment (four patients had <0.1 and one >3.6) and nine patients on LT3 treatment (three patients had TSH level <0.1 mU/L and six >3.6). TSH levels were significantly higher in LT3 group compared to LT4 group (LT3: median 1.33 mU/L (IQR 0.47-2.26) *vs*. LT4: median 0.61 mU/L (IQR 0.25-1.20); *P*<0.018).

### D2-Thr92Ala Polymorphisms

We identified the D2-Thr92Ala deiodinase 2 polymorphism in 61.0% of the patients: 32 (54.2%) were heterozygous for D2-Thr92Ala deiodinase 2 and four (6.8%) were homozygous.

A one-way ANOVA was conducted to compare the effect of the D2-Thr92Ala polymorphism on ThyPRO scale scores for both LT4 and LT3 treatment. There was a significant difference in only one ThyPRO scale score (overall QoL) among patients receiving LT4 treatment, where D2-Thr92Ala homozygous patients exhibited significantly lower scores (*P*=0.041).

### QoL Questionnaires

#### Fatigue Questionnaire

**Figure 2:**
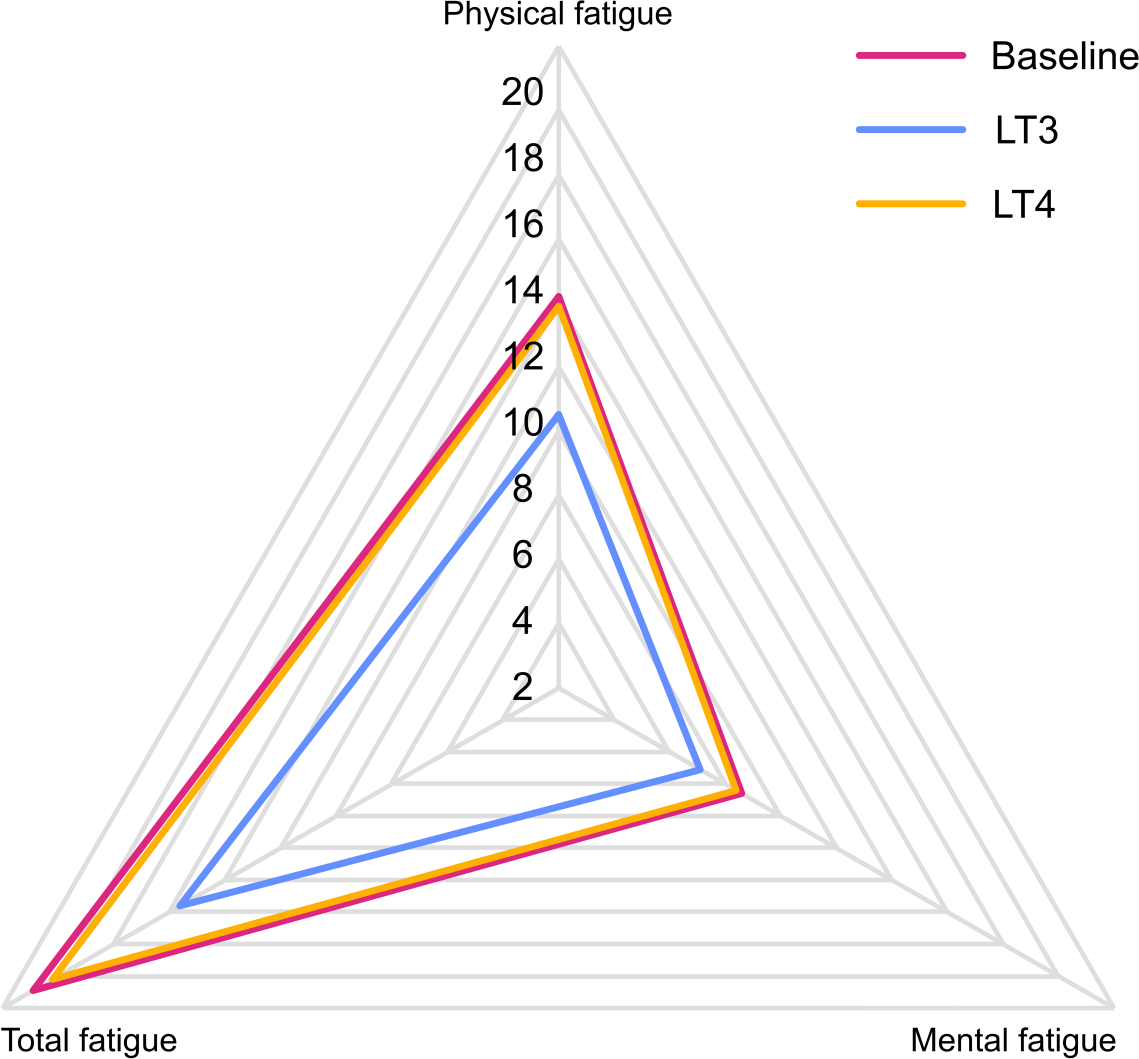
Fatigue Questionnaire radar plot. Radar plot of the Fatigue Questionnaire dimension scores at baseline and after 12 weeks treatment periods with LT4 and LT3, respectively. Higher scores imply higher levels of fatigue.

There was a statistically significant difference between scores in all three dimensions (physical, mental and total fatigue) of the Fatigue Questionnaire after 12 weeks of treatment with LT3 compared to baseline, with lower scores after LT3 treatment, indicating less fatigue (**Table 2** and **Figure 2**). No differences in either of the three fatigue dimension scores after 12 weeks treatment with LT4 compared to baseline were demonstrable. All three dimensions’ scores were also significantly better when comparing LT3 with LT4 treatment groups.

**Table 2:** Fatigue Questionnaire dimension scores.

	Baseline (n = 59)	On LT4 (n = 49)		On LT3 (n = 48)		LT4 *vs*. LT3 (n = 47)	
	Score	Change from baseline	*P* value	Change from baseline	*P* value	Change from LT4 to LT3	*P* value
Physical fatigue	12.2 ± 3.9	-0.5 ± 4.7	0.438	-4.0 ± 6.2	<0.001	-3.2 ± 6.1	0.001
Mental fatigue	6.6 ± 2.4	-0.4 ± 2.4	0.203	-1.8 ± 2.9	<0.001	-1.2 ± 2.7	0.004
Total fatigue	18.9 ± 5.8	-1 ± 6.6	0.303	-5.8 ± 8.7	<0.001	-4.4 ± 8.3	0.001

Data are presented as mean ± SD. Combining the scores of physical and mental fatigues produce total fatigue, with a maximum score of 33. Higher scores imply higher levels of fatigue.

#### SF-36

**Figure 3:**
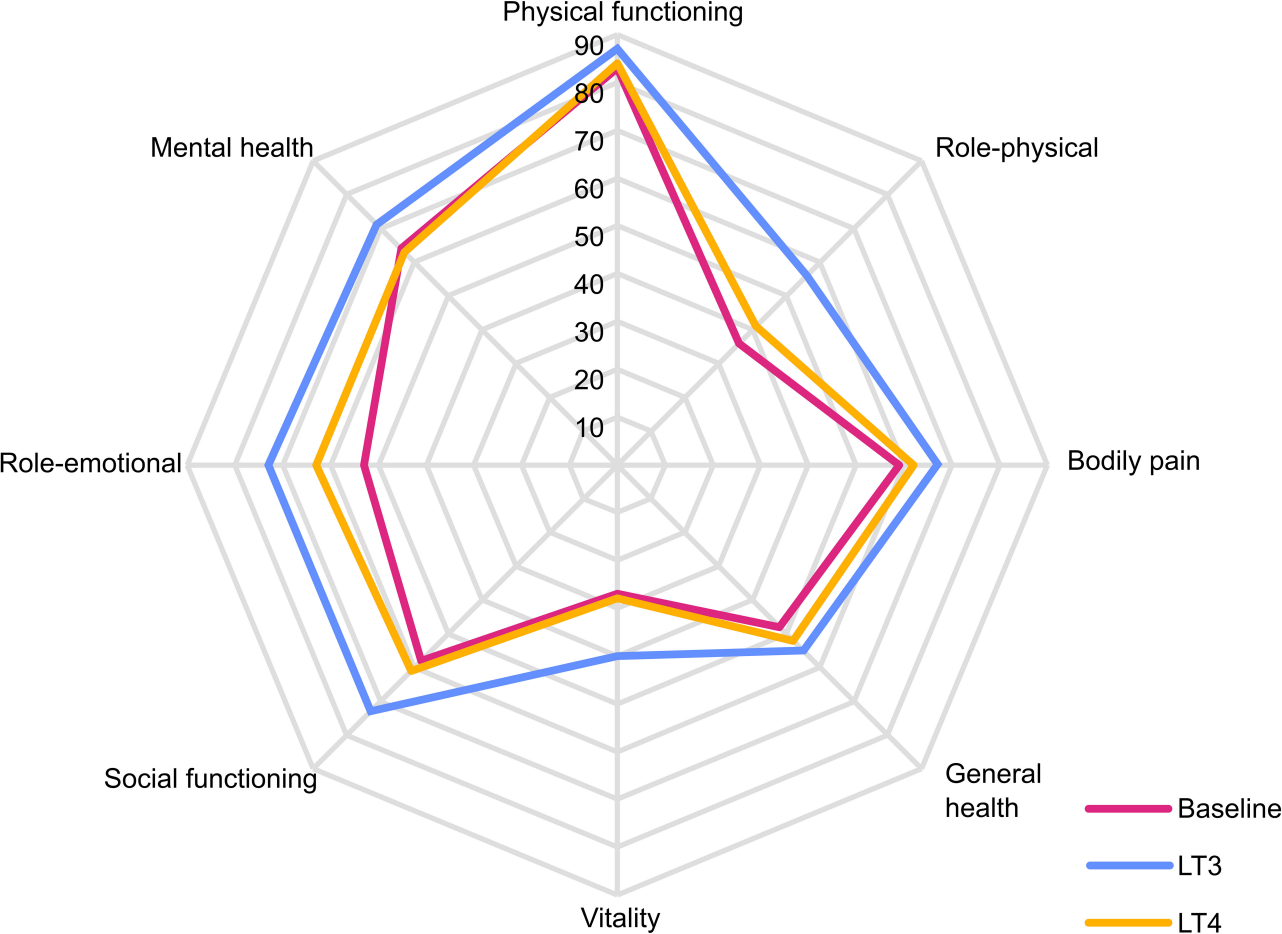
SF-36 radar plot. Radar plot of SF-36 scale scores at baseline and after 12 weeks treatment periods with LT4 and LT3, respectively. Each scale ranges 0-100, with higher scores indicating better quality of life.

All SF-36 scale scores except one (bodily pain) were significantly higher after 12 weeks of LT3 treatment compared to baseline, indicating improved QoL (**Table 3** and **Figure 3**). None of the scale scores changed between baseline and LT4 treatment. However, when comparing LT4 and LT3 treatment periods, only three scales were significantly higher in the LT3 group (vitality, social functioning, and mental health).

**Table 3:** SF-36 scale scores.

	Baseline (n = 59)	On LT4 (n = 49)		On LT3 (n = 48)		LT4 *vs*. LT3 (n = 47)	
	Score	Change from baseline	*P* value	Change from baseline	*P* value	Change from LT4 to LT3	*P* value
Physical functioning	83 ± 14	1 ± 11	0.379	4 ± 12	0.018	3 ± 12	0.121
Role-physical	36 ± 35	4 ± 35	0.415	19 ± 40	0.002	13 ± 32	0.08
Bodily pain	59 ± 23	3 ± 22	0.394	6 ± 23	0.067	3 ± 21	0.305
General health	48 ± 20	4 ± 16	0.098	6 ± 16	0.011	2 ± 16	0.525
Vitality	27 ± 14	3 ± 16	0.257	14 ± 22	<0.001	11 ± 21	0.001
Social functioning	58 ± 21	1 ± 25	0.726	14 ± 29	0.002	11 ± 24	0.004
Role-emotional	53 ± 42	7 ± 39	0.182	20 ± 44	0.003	11 ± 37	0.058
Mental health	64 ± 16	-2 ± 15	0.439	7 ± 15	0.003	8 ± 14	<0.001

Data are presented as mean ± SD. Each scale in SF-36 is scored between 0-100, where higher scores indicating better QoL.

#### ThyPRO

**Figure 4:**
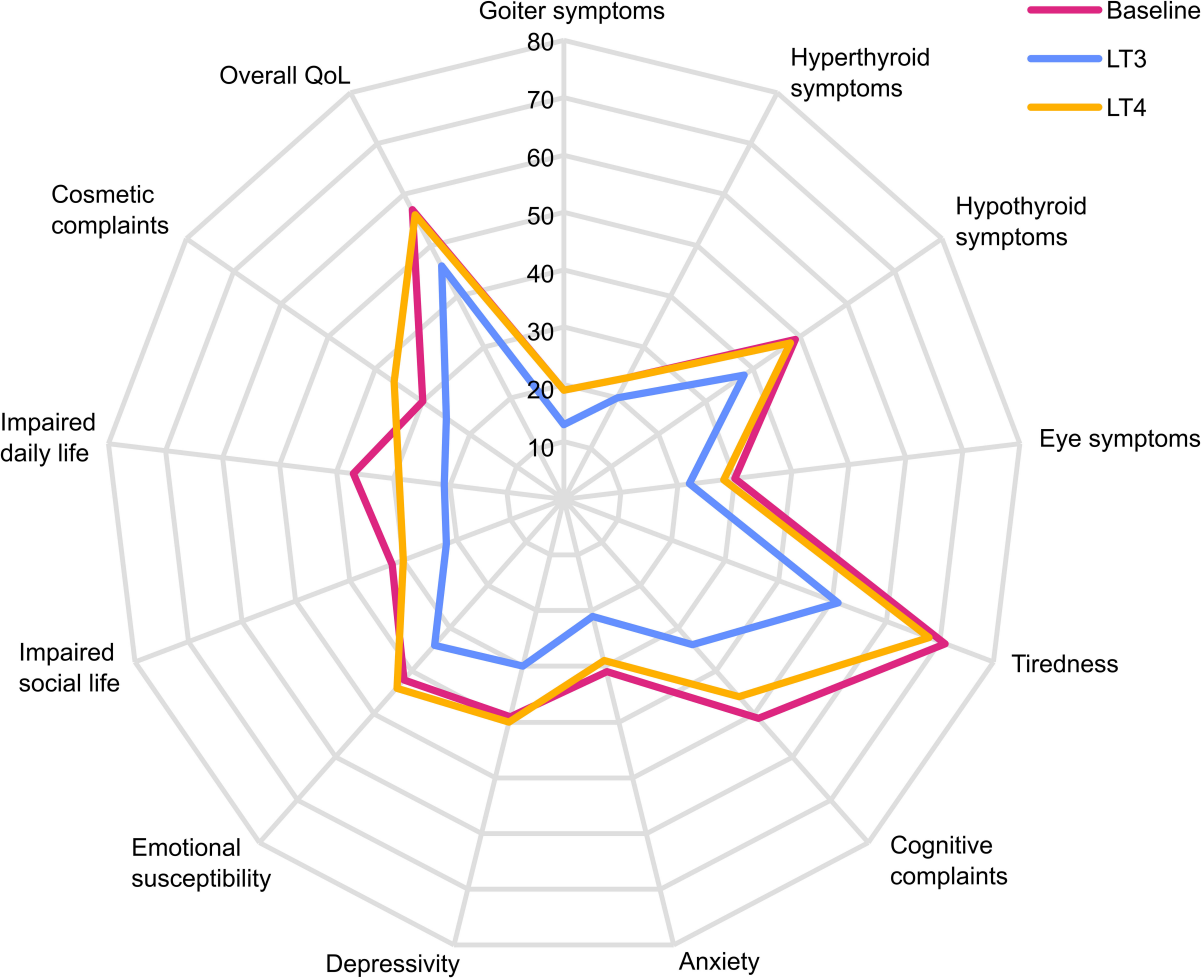
ThyPRO radar plot. Radar plot of ThyPRO scale scores at baseline and after 12 weeks treatment periods with LT4 and LT3, respectively. Each scale ranges 0-100, with higher scores indicating poorer quality of life.

Compared to baseline, 12 weeks of treatment with LT3 led to significantly lower scores in all ThyPRO scale scores except one (cosmetic complaints). The most pronounced effect was less tiredness (-21 ± 26; *P*<0.0001) and less cognitive complaints (-20 ± 20; *P*<0.0001) (**Table 4** and **Figure 4**). During LT4 treatment we found significantly lower scores in two ThyPRO scales (cognitive complaints and impaired daily life) compared to baseline. When comparing LT3 and LT4 treatment groups, all ThyPRO scale scores were significantly lower, indicating improved QoL, except in the hyperthyroid symptoms scale and eye symptoms scale. These two scales showed also lower scores on LT3, but the differences were not significant.

**Table 4:** ThyPRO scale scores.

	Baseline (n = 59)	On LT4 (n = 49)		On LT3 (n = 48)		LT4 *vs*. LT3 (n = 47)	
	Score	Change from baseline	*P* value	Change from baseline	*P* value	Change from LT4 to LT3	*P* value
Goiter symptoms	19 ± 18	1 ± 17	0.730	-5 ± 12	0.014	-6 ± 14	0.030
Hyperthyroid symptoms	24 ± 15	-1 ± 13	0.493	-5 ± 13	0.008	-4 ± 14	0.081
Hypothyroid symptoms	49 ± 20	-2 ± 19	0.417	-12 ± 19	<0.001	-9 ± 19	0.002
Eye symptoms	30 ± 20	-3 ± 16	0.202	-8 ± 15	0.001	-4 ± 16	0.073
Tiredness	71 ± 16	-4 ± 19	0.110	-21 ± 26	<0.001	-15 ± 27	<0.001
Cognitive complaints	51 ± 26	-9 ± 20	0.003	-20 ± 20	<0.001	-9 ± 22	0.006
Anxiety	31 ± 25	1 ± 19	0.636	-9 ± 24	0.008	-8 ± 18	0.004
Depressivity	39 ± 21	0 ± 19	0.968	-10 ± 21	0.003	-9 ± 18	0.001
Emotional susceptibility	42 ± 22	0 ± 21	0.881	-9 ± 24	0.018	-8 ± 19	0.004
Impaired social life	32 ± 21	-3 ± 19	0.343	-10 ± 21	0.002	-7 ± 18	0.015
Impaired daily life	37 ± 23	-9 ± 18	0.001	-16 ± 23	<0.001	-7 ± 22	0.041
Cosmetic complaints	30 ± 23	7 ± 19	0.022	-3 ± 20	0.275	-9 ± 16	<0.001
Overall QoL	57 ± 25	-1 ± 27	0.892	-11 ± 29	0.017	-9 ± 28	0.041

Data are presented as mean ± SD. Each scale in ThyPRO is scored between 0-100, where higher scores indicating worse QOL.

### Safety Parameters

No significant differences were seen in cardiovascular effects as reflected by resting heart rate (LT3: mean 66.4 bpm ±10.3 *vs*. LT4: mean 65.0 bpm ±8.7; *P*=0.169), systolic (LT3: mean 117.1 mmHg ± 10.9 *vs*. LT4: mean 117.6 mmHg ± 11.2; *P*=0.832) or diastolic (LT3: mean 77.3 mmHg ±6.0 *vs*. LT4: mean 77.3 mmHg ±5.4; *P*=0.641) blood pressure after LT3 treatment compared to LT4 treatment (**Table 5**).

**Table 5:** Safety parameters.

	Baseline (n = 59)	After 12 weeks LT4 (n = 49)		After 12 weeks LT3 (n = 48)		LT4 *vs*. LT3 (n = 47)
	Result	Result	*P* value	Result	*P* value	*P* value
Resting heart rate (beats per minute)	64.7 ± 11.2	65.0 ± 8.7	0.869	66.4 ± 10.3	0.079	0.169
Systolic blood pressure (mmHg)	118.0 ± 13.7	117.6 ± 11.2	0.524	117.1 ± 10.9	0.227	0.832
Diastolic blood pressure (mmHg)	78.3 ± 7.9	77.3 ± 5.4	0.064	77.3 ± 6.0	0.100	0.641

Data are presented as mean ± SD.

### Patients’ Preference

At the end of our study, we asked all 47 patients who completed both treatment periods which treatment they preferred. Sixteen (34%) patients preferred LT4, 28 (60%) preferred LT3, while 3 (6%) had no preference.

## Discussion

This is the first crossover study evaluating the effects of liothyronine versus levothyroxine on quality of life in female patients with residual hypothyroid symptoms, despite adequate hormonal substitution with LT4. Our results show that patients on LT3 monotherapy experienced significant better QoL as assessed by both thyroid-specific and generic QoL questionnaires compared to patients on LT4 treatment.

Goiter and hypothyroid symptoms, tiredness, cognitive complaints, anxiety, depressivity, emotional susceptibility, impaired social and daily life, cosmetic complaints and overall quality of life were obtained from the ThyPRO questionnaire and showed significantly better results after 12 weeks on LT3 therapy compared to LT4. There were no differences, however, in hyperthyroid and eye symptoms between the treatment groups.

Fatigue was reported among more than 95% of our patient population despite optimal LT4 therapy. The findings from the analysis of the Fatigue Questionnaire with significantly better results in all three dimensions (physical, mental and total fatigue) show that LT3 therapy indeed improved this prevalent symptom compared to LT4. It is also noteworthy that none of the scales or dimensions in any of the three questionnaires showed poorer outcomes on LT3 compared to LT4 treatment. Analysis of patients’ preference showed that almost two-thirds of the patients who completed the study preferred LT3 treatment.

Our findings are consistent with the results previously described by two research groups reporting superior results on a combination therapy with LT4 and LT3 (8–11). In particular, compared to normative SF-36 data, treatment with LT3 improved QoL in the hypothyroid patients in our study to levels that approach the general Norwegian population reported by Loge et al. (32).

Five out of six patients who withdrew from our study due to worsening of symptoms, were in the LT3 treatment group. A reason may be that the short half-life of LT3 cause temporary side effects with hyperthyroid symptoms. It is also possible, that only a selected group of hypothyroid patients will experience improvement in QoL on LT3 monotherapy due to differences in T3 production from an impaired thyroid gland. Our study was not powered for analyses of such sub-groups.

No significant changes in resting heart rate or blood pressure were registered in patients on LT3. Since cardiovascular disease was an exclusion criterion, this may explain the absence of cardiovascular adverse events. It is also possible that the average age of 42.9 years among study patients is underestimating cardiac side effects from LT3 therapy that might occur in an older patient population. Therefore, we cannot exclude an increased risk of cardiovascular events on LT3 treatment compared to LT4 among older age groups. Furthermore, the short treatment period of 12 weeks might not have been long enough for side-effects to occur. In addition, another limitation in the assessment of cardiovascular function, is the lack of an ECG examination, ideally a 24h ECG, after the two treatment periods. During physical examinations, however, we did not record any cases of irregular pulse.

The D2-Thr92Ala polymorphism has a reported prevalence of 10.7% as homozygous carriers in the general population and 11.3% in hypothyroid patients on LT4 treatment (33). In the present study, we identified the homozygous variant of this polymorphism in only 6.8% of our study patients. This relatively low proportion of individuals may not be representative of other populations. Paradoxically, the only significant difference we found between the presence of D2-Thr92Ala polymorphism and a non-Thr92Ala polymorphism in the deiodinase 2 gene, was a lower score in overall QoL on LT4 treatment, indicating improved QoL. This result is in contrast to the hypothesis that this polymorphism may impair the effect of deiodinase 2 and reduce the conversion of T4 to T3 at the cellular level. However, since none of the other ThyPRO scales showed any associations between type of polymorphism and score achieved, and the study was not adequately powered to look at the impact of polymorphism, this finding should be interpreted with caution.

This study has several strengths. First, we evaluated QoL by three different questionnaires, both generic and disease-specific, which strengthens the result with demonstrable improvements of QoL remaining consistent across all questionnaires. Furthermore, we achieved euthyroidism in patients treated with LT3, contrary to some previous studies on combination LT4/LT3 therapy, where many patients were hyperthyroid (8, 10, 34–36). At last, our main inclusion criterion was patients with residual hypothyroid symptoms because our aim was to evaluate whether this group of patients presented better QoL on LT3 compared to LT4. This is the group of patients where discussions concerning alternatives to LT4 monotherapy are relevant, as also emphasized by ATA (37). By evaluating this group, the result is not affected by hypothyroid patients that are asymptomatic on LT4 in monotherapy and who might potentially experience deterioration of QoL on LT3 monotherapy due to side effects. However, one limitation of this study is the fact that a validated questionnaire was not used to examine the presence of residual hypothyroid symptoms at inclusion.

Our results are in contrast to the study by Celi et al. who reported that there was no difference in QoL between LT3 and LT4 monotherapy (24). However, persistent hypothyroid symptoms were not an inclusion criterion in that study, QoL was assessed using only two questionnaires and follow-up time was only approximately 30 days (24).

The main limitation of the present study was that the patients were not blinded to treatment which may have led to skewed results. We cannot rule out that the observed improvements in QoL were due to the patients´ knowledge of treatment and expectations. We certainly considered a blinded study initially. However, in order to meet the requirements for blinding in a study where treatment doses are individualized, given thrice daily and potential adjustments are made every four weeks, it would be necessary with a comprehensive logistics setup including custom made tablets at great cost. Unfortunately, we had neither available to the extent required. Since each patient was treated with an individual dose of study treatment (both LT4 and LT3) based on earlier treatment dose and TSH levels, and each dose was evaluated for adjustment every four weeks, it would have been necessary for some patients to take many tablets each day to keep the study blinded to the patients. In similar studies, each patient had to take up to six tablets at each dose (23). Furthermore, we are unconvinced that blinding could be complete. Because of the differences in half-life of approximately one day for LT3 (38) and seven days for LT4 (39), we argue that in a blinded study, patients would nevertheless notice the difference between the two treatments. Thus, they could still be biased anyway, resulting in false positive results. Despite the lack of blindness and due to the pronounced differences recorded between LT3 and LT4 therapy, we would claim the importance in conducting this study. However, future blinded, randomized and controlled trials on LT3 monotherapy and QoL need to be performed to support our findings.

In conclusion, treatment with LT3 monotherapy increased QoL compared to LT4 without inducing biochemical or clinical hyperthyroidism or adverse cardiovascular effects. This is supported by consistent results indicating improvement obtained from disease-specific and generic questionnaires. Treatment with LT3 monotherapy may therefore be an experimental treatment option in hypothyroid patients who suffer from residual symptoms despite LT4 treatment. However, long-term studies are needed to assess the long-term safety of this therapy regimen. Five patients on LT3 dropped out of the study due to side effects, which may indicate that some patients do not seem to tolerate LT3 monotherapy. Based on our results, the D2-Thr92Ala polymorphism was not associated with reduced thyroid-specific QoL or treatment effects.

## Data Availability Statement

The raw data supporting the conclusions of this article will be made available by the authors, without undue reservation.

## Ethics Statement

The studies involving human participants were reviewed and approved by The Regional committee for ethics in medical research in Norway. The patients/participants provided their written informed consent to participate in this study.

## Author Contributions

Conceived and designed the experiments: BAB, SSH, HLG, and EFE. Performed the experiments: BAB and SLØ. Contributed materials/analysis tools: LJO and TJB. Wrote the paper: BAB, SSH, HLG, TJB, LJO, SLØ, and EFE. All authors contributed to the article and approved the submitted version.

## Funding

This work was supported by research funding from the South-Eastern Norway Regional Health Authority (2017101).

## Conflict of Interest

Authors SSH and EFE were employed by Pilestredet Park Specialist Center and LJO was employed by Balderklinikken.

The remaining authors declare that the research was conducted in the absence of any commercial or financial relationships that could be constructed as a potential conflict of interest.

## Publisher’s Note

All claims expressed in this article are solely those of the authors and do not necessarily represent those of their affiliated organizations, or those of the publisher, the editors and the reviewers. Any product that may be evaluated in this article, or claim that may be made by its manufacturer, is not guaranteed or endorsed by the publisher.
