# Supplementary material for: Effect of Liothyronine Treatment on Quality of Life in Female Hypothyroid Patients With Residual Symptoms on Levothyroxine Therapy: A Randomized Crossover Study
Source: Front Endocrinol (Lausanne). 2022 Feb 22;13:816566. doi: 10.3389/fendo.2022.816566 (PMC8902821; doi:10.3389/fendo.2022.816566)
Supplement: Supplementary file 1 [file DataSheet_1.docx]

| Residual hypothyroid symptoms despite adequate LT4 or LT4/LT3 combination therapy: | | |
| --- | --- | --- |
| Symptom | | \| Start date  (month - year) \| \| \| \| \| \| \| \| --- \| --- \| --- \| --- \| --- \| --- \| --- \| \| M \| M \| - \| Y \| Y \| Y \| Y \| |
|  | |  |
| Cold-intolerance | Yes No | \|  \|  \| - \|  \|  \|  \|  \| \| --- \| --- \| --- \| --- \| --- \| --- \| --- \| |
| Fatigue | Yes No | \|  \|  \| - \|  \|  \|  \|  \| \| --- \| --- \| --- \| --- \| --- \| --- \| --- \| |
| Cognitive disturbances | Yes No | \|  \|  \| - \|  \|  \|  \|  \| \| --- \| --- \| --- \| --- \| --- \| --- \| --- \| |
| Emotional disturbances | Yes No | \|  \|  \| - \|  \|  \|  \|  \| \| --- \| --- \| --- \| --- \| --- \| --- \| --- \| |
| Edemas | Yes No | \|  \|  \| - \|  \|  \|  \|  \| \| --- \| --- \| --- \| --- \| --- \| --- \| --- \| |
| Dry skin | Yes No | \|  \|  \| - \|  \|  \|  \|  \| \| --- \| --- \| --- \| --- \| --- \| --- \| --- \| |
| Menstrual disorders | Yes No | \|  \|  \| - \|  \|  \|  \|  \| \| --- \| --- \| --- \| --- \| --- \| --- \| --- \| |
| Weight gain | Yes No | \|  \|  \| - \|  \|  \|  \|  \| \| --- \| --- \| --- \| --- \| --- \| --- \| --- \| |
| Hair loss | Yes No | \|  \|  \| - \|  \|  \|  \|  \| \| --- \| --- \| --- \| --- \| --- \| --- \| --- \| |
| Obstipation | Yes No | \|  \|  \| - \|  \|  \|  \|  \| \| --- \| --- \| --- \| --- \| --- \| --- \| --- \| |
